# Photothermal-Induced Antibacterial Activity of Gold Nanorods Loaded into Polymeric Hydrogel against *Pseudomonas aeruginosa* Biofilm

**DOI:** 10.3390/molecules24142661

**Published:** 2019-07-23

**Authors:** Amal G. Al-Bakri, Nouf N. Mahmoud

**Affiliations:** 1 School of Pharmacy, The University of Jordan, Amman 11942, Jordan; 2 Faculty of Pharmacy, Al-Zaytoonah University of Jordan, Amman 11733, Jordan

**Keywords:** photothermal therapy, phospholipid, gold nanorods, *Pseudomonas aeruginosa*, biofilm, hydrogel

## Abstract

In this study, the photothermal-induced bactericidal activity of phospholipid-decorated gold nanorods (DSPE-AuNR) suspension against *Pseudomonas aeruginosa* planktonic and biofilm cultures was investigated. We found that the treatment of planktonic culture of *Pseudomonas aeruginosa* with DSPE-AuNR suspension (0.25–0.03 nM) followed by a continuous laser beam exposure resulted in ~6 log cycle reduction of the bacterial viable count in comparison to the control. The percentage reduction of *Pseudomonas aeruginosa* biofilm viable count was ~2.5–6.0 log cycle upon laser excitation with different concentrations of DSPE-AuNR as compared to the control. The photothermal ablation activity of DSPE-AuNR (0.125 nM) loaded into poloxamer 407 hydrogel against *Pseudomonas aeruginosa* biofilm resulted in ~4.5–5 log cycle reduction in the biofilm viable count compared to the control. Moreover, transmission electron microscope (TEM) images of the photothermally-treated bacteria revealed a significant change in the bacterial shape and lysis of the bacterial cell membrane in comparison to the untreated bacteria. Furthermore, the results revealed that continuous and pulse laser beam modes effected a comparable photothermal-induced bactericidal activity. Therefore, it can be concluded that phospholipid-coated gold nanorods present a promising nanoplatform to eradicate *Pseudomonas aeruginosa* biofilm responsible for common skin diseases.

## 1. Introduction

Biofilms are functional consortia of sessile microorganisms encased within a self-generated extracellular polymeric matrix composed of polysaccharides; proteins and DNA [1,2]. Biofilms exhibit distinct architectural; phenotypic and biochemical characteristics [3,4] that give them advantages over their planktonic counterparts in terms of virulency; pathogenicity and resistance to antimicrobial agents [1,4].

The biofilm trait of high antimicrobial resistance to antibiotics and disinfectants is a multifactorial and is attributed to slow antibiotic penetration, reduced microbial growth rates, persisters and unique physiology [5,6].

Essentially, biofilm-derived infections are aggressive, persistent and difficult to treat. *Pseudomonas aeruginosa* is a versatile opportunistic bacterial pathogen [7] responsible for a wide array of life-threatening acute and chronic infections [8]. *Pseudomonas aeruginosa* is among the most common colonizers of infected wounds and is an abundant biofilm former [9]. Biofilms are a protected mode of growth presenting a major problem in infection treatment due to high recalcitrance to chemotherapy treatment [10]. Within a chronic wound, the biofilm tolerance to several antibiotics and disinfectants causes the failure of commonly used chemotherapy to reduce biofilm wound bioburden, and consequently, impedes the wound healing cascade [11].

The antimicrobial treatment of *Pseudomonas aeruginosa* wound infections is challenged by its characteristic intrinsic, acquired and biofilm mode of resistance [12,13,14,15].

Unfortunately, failure in treatment is associated with septicaemia which is a fatal complication. Due to this, new alternative non-antibiotic methods should be explored for success in controlling and eradicating *Pseudomonas aeruginosa* biofilm-based infections.

Gold nanoparticles (AuNP), and in particular, gold nanorods (AuNR), have received marked interest in nanomedicine and drug delivery, due to their exclusive optical properties and the ease with which their surfaces can be functionalized with diverse ligands and moieties [16,17]. Upon near infra-red (NIR) excitation into the confined surface plasmon resonance bands of AuNR, the excited electrons release their energy as localized heat [18,19], and this photothermal effect is responsible for the intriguing therapeutic applications of AuNR, such as photothermal ablation of cancer cells [20] and eradication of several pathogenic micro-organisms [21,22]. Therefore, nanomaterial-based antibacterial platforms are considered a smart solution for bacterial infections’ management since therapeutic failure due to resistant bacterial strains and biofilm formation presents a major health challenge.

The bacterial uptake of gold nanoparticles, which is essential for their photothermal ablation activity, is highly dependent on the size and surface chemistry of the nanoparticles. We previously investigated the bactericidal effect and photothermal-based antibacterial activity of gold nanorods that contain several surfaces decorating ligands against common skin pathogens [21,22,23]. The results revealed that the surface functionality of the nanoparticles has a key role in enhancing the uptake and destructive ability of AuNP towards bacteria. For example, phospholipid-decorated AuNR demonstrated potent bactericidal activity against *Staphylococcus aureus* as compared to AuNR coated with cholesterol moiety [22].

The photothermal-based bacterial eradicating activity of AuNP was demonstrated in several studies, either independently or in conjugation with drugs [24,25,26]. In one study, AuNPs resulted in around a 50% reduction in Candida albicans biofilm [27], and in another study, gold nanocrosses were targeted against the *Pseudomonas aeruginosa* biofilm using antibodies, that resulted in complete eradication of the biofilm upon NIR excitation with an 800 nm laser beam at a power density of ~3.0 W cm^−2^ [28]. The photothermal-based antibacterial activity of monolayers of gold nanostars against planktonic and biofilm bacteria was investigated by Pallavicini, et al. [29,30]. Additionally, the antibacterial activity of photo-thermally active Prussian blue nanoparticles has been reported recently [31].

Hydrogels have biocompatible and biodegradable characteristics which make them an attractive vehicle for topical delivery systems [32,33,34,35]. Poloxamer 407 (Pluronic^®^ F127) is a non-ionic surfactant characterized by thermo-reversible gelation activity, rendering it an attractive formulation choice for many drug delivery systems [36,37,38]. Poloxamer 407 aqueous solutions are fluid below the sol-gel transition temperature (T sol-gel), and the solutions turn to a semi-solid state above this temperature [36]. Significantly, poloxamer 407 polymer was used in our current study as a vehicle of the nanorods to enhance their application and improve their contact duration and deposition within a bacterial biofilm. Furthermore, it demonstrated an accelerated wound healing ability through the enhancement of cell proliferation and production of epidermal growth factor, in addition to its anti-microbial activity [36,39,40]. Recently, in an experiment, AuNP loaded into poloxamer 407 hydrogel demonstrated an enhanced scar-less healing of the wound in rats and accelerated collagen deposition at the wound site [41].

In this study, gold nanorods decorated with phospholipid-polyethylene glycol (PEG) were prepared, characterized and loaded into poloxamer 407 hydrogel. The photothermal-induced antibacterial activity of the surface-modified gold nanorods of different concentrations was evaluated against *Pseudomonas aeruginosa* planktonic and biofilm culture. Bactericidal activity against *Pseudomonas aeruginosa* biofilm was investigated for AuNR upon loading into poloxamer 407 hydrogel using continuous and pulsed laser modality. Fluorescence microscopy and transmission electron microscope (TEM) were used to characterize the photothermal ablation effect of DSPE-AuNR against *Pseudomonas aeruginosa*.

## 2. Results and Discussion

Chronic skin wounds represent a major health burden associated with high morbidity and mortality due to susceptibility to infection.

In this study, photothermal-induced antibacterial activity was investigated for phospholipid-coated AuNR against *Pseudomonas aeruginosa* planktonic and biofilm cultures. The effect of continuous *vs*. pulse modes of laser treatment on bacterial photothermal ablation was examined. Furthermore, the photothermal ablation of DSPE-AuNR suspended in poloxamer gel against *Pseudomonas aeruginosa* was also evaluated.

### 2.1. Synthesis and Characterization of AuNR Suspensions

**Figure 1:**
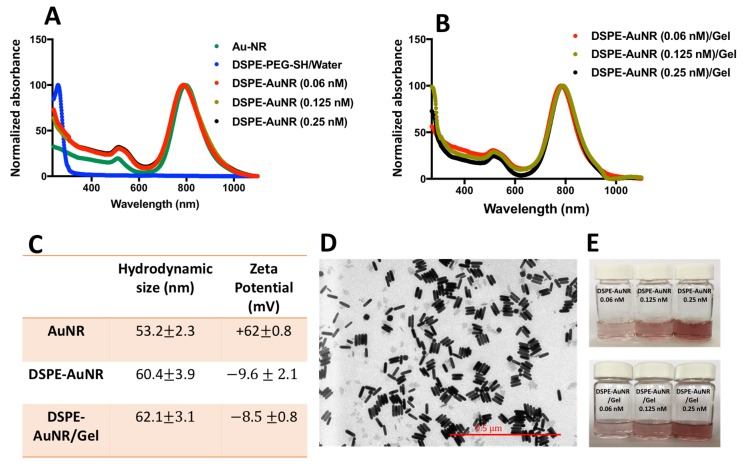
Characterization of AuNP and DSPE-AuNR suspensions. (**A**) UV-Vis spectra of CTAB-coated-AuNR and upon surface replacement of CTAB by DSPE-PEG-SH ligand (at different molar concentrations). (**B**) UV-Vis spectra of AuNR suspension of different molar concentrations (0.06, 0.125 and 0.25 nM) loaded into poloxamer 407 hydrogel. (**C**) Hydrodynamic size measured by DLS and zeta potential of AuNR, DSPE-AuNR suspensions and DSPE-AuNR loaded into hydrogel. (**D**) TEM image of DSPE-AuNR suspension confirms the rod shape of the nanoparticles having an average AR of 3.88. (**E**) Photos of DSPE-AuNR suspension and DSPE-AuNR loaded into poloxamer 407 hydrogel at different molar concentrations which confirms the colloidal stability of the nanorods on being loaded into the hydrogel.

AuNR were synthesized as described previously [42] and characterized by optical spectroscopy, zeta potential and hydrodynamic size to confirm successful surface functionalization. Figure 1A demonstrates the normalized optical absorption spectra of AuNR of different concentrations (0.06, 0.125 and 0.25 nM) with typical transverse and longitudinal peaks at 520 nm and 805 nm, respectively without broadening or tailing. The attachment of thiolated ligand to the surface of AuNR occurs through the coordinate bond between gold and the thiol group of DSPE-PEG-SH [43,44,45]. The optical spectrum of DSPE-PEG-SH ligand dispersed in water revealed a peak at 265 nm. Upon functionalization of AuNR with DSPE-PEH-SH ligand, a new peak was observed in the spectrum of DSPE-AuNR at 265 nm, confirming the successful attachment of DSPE-PEG-SH to the surface of AuNR. The hydrodynamic diameter of the nanorods measured through dynamic light scattering (DLS) was increased upon surface decoration of the nanorods with DSPE-AuNR. In addition, the effective surface charge of AuNR was shifted from positive zeta potential due to the surface absorbed cetyltrimethylammonium bromide (CTAB) molecules to slightly negative charge (−10 mV) upon functionalization with DSPE-PEG-SH, which confirms the successful functionalization with DSPE-PEG-SH (Figure 1C).

The loading of AuNR suspension of different molar concentrations (0.06, 0.125 and 0.25 nM) into poloxamer 407 hydrogel gel did not affect the colloidal stability of the nanorods as no change in their colloidal colour was observed upon loading into the hydrogel. Furthermore, the normalized optical spectra of DSPE-AuNR loaded into the hydrogel revealed no significant broadening or tailing of the longitudinal plasmon peak (Figure 1B) which confirms their excellent colloidal stability. Moreover, no significant changes were observed in the hydrodynamic size and zeta potential of the nanorods upon loading into the hydrogel which suggests their excellent colloidal stability (Figure 1C). The sol-gel transition temperature of DSPE-AuNPs loaded into the hydrogel was ~35.9 °C at 18% of poloxamer 407, suggesting that the prepared hydrogel of AuNR could be converted into gel form at body temperature.

The shape and size of the nanorods were characterized by transmission electron microscope (TEM). Figure 1D shows the TEM image of DSPE-AuNR which confirms the rod shape of the nanoparticles with an average width and length of 49.8 ± 2.6 nm and 11.8 ± 1.8 nm respectively and an average aspect ratio (AR) of 3.88. As presented in Figure 1E, the colloidal colour of DSPE-AuNR loaded into poloxamer 407 hydrogel is compared to the original colloidal colour of the gold nanorods, confirming the excellent colloidal stability of the nanorods upon loading into the hydrogel at different molar concentrations. The surface functionalization of AuNR by DSPE-PEG-SH ligand was validated previously by ^1^H-NMR in our recent work [22].

### 2.2. Antibacterial Activity of DSPE-AuNR Suspension against Pseudomonas Aeruginosa

The antibacterial activity of DSPE-AuNR against planktonic *Pseudomonas aeruginosa* culture was evaluated by determining the minimum inhibitory concentration (MIC) and the equivalent bactericidal effect at one concentration within the MIC range. The MIC value of DSPE-AuNR suspension was 0.75 nM (~65.5 μg/mL) which resulted in 99.998% reduction in bacterial viability (~4.7-Log cycle). As indicated in the literature, AuNP of different sizes, shapes and surface chemistries have antibacterial activity at the concentration range of ~10–200 μg/mL [46]. Compared to silver nanoparticles, Salomoni, et al., reported three log cycle reduction of *Pseudomonas aeruginosa* viability upon treatment with silver nanoparticles at the concentration 5.0 μg/mL [47].

In order to exclude the possible contribution of residual CTAB or the coating material on the observed antibacterial of AuNR, the MIC was performed for the supernatant of DSPE-PEG-GNR after centrifugation and for the aqueous dispersion of DSPE-PEG-SH ligand. No antibacterial activity was observed for the supernatant of DSPE-AuNR suspension after centrifugation and for the aqueous dispersion of DSPE-PEG-SH, eliminating the possible contribution of CTAB or the coating material on the antibacterial activity of DSPE-AuNR.

In our recent study, we demonstrated that PEG-AuNR at 4.0 nM effected only 2.0 log cycle reduction in bacterial viability and no significant antibacterial activity was observed for PEG-AuNR at 0.75 nM [41]. In this study, AuNR were functionalized with a thiolated-PEGylated phospholipid moiety which is supposed to enhance their colloidal stability and bacterial uptake and maximize their photothermal effect at a low concentration.

Poor outer membrane permeability of *Pseudomonas aeruginosa* limits the activity of several antibiotics [48]. To this end, lipid-based nanocarriers were demonstrated to enhance the cellular uptake of the loaded drug through endocytosis [49] or destabilizing membranes by means of physicochemical interactions with membrane components. For example, phospholipids can have a pivotal role in destabilizing the outer membrane of Gram-negative bacteria and thus increase its permeability due to its inherent high affinity towards Ca2+ and Mg2+ membrane bound ions [50]. Essentially, the phospholipids, 1-palmitoyl-2-hydroxy-sn-glycero-3-phosphate phospholipid drastically enhanced the permeability of β-lactam antibiotics through *Pseudomonas aeruginosa* [51]. Moreover, Baker, et al., found that exposing *Pseudomonas aeruginosa* to exogenous fatty acids enhanced the bacterial membrane permeation and decreased their resistance towards polymyxin B by inducing significant changes in the bacterial membrane phospholipid structure [52]. Furthermore, the antibacterial activity of monoglycerides-conjugated lipid nanocapsules was reported against *Staphylococcus aureus* and methicillin-resistant *Staphylococcus aureus* [53]. Based on these previous results, we synthesized a phospholipid-coated AuNR as a promising ligand to be assessed for antibacterial activity. In our recent work, we demonstrated that the phospholipid-based nanogold suspension shows potent bactericidal activity (~0.011 nM) against the Gram-positive *Staphylococcus aureus* [22].

It is essential to mention that our nanorods were found to be stable when mixed with bacterial growth medium, and there was no significant aggregation of the nanorods as evidenced by their colloidal colour and optical spectra (results not shown). The colloidal stability of AuNR upon exposure to biological barrier or bacterial growth medium was found to depend greatly on nanoparticles’ surface functionalization [23,54]. Exposure of AuNP to biological barriers or growth media induced nanoparticles aggregation and severely influenced their biological activity [23,54].

### 2.3. Photothermal-Based Antibacterial Activity of DSPE-AuNR Suspension Against Pseudomonas Aeruginosa

Photothermal-based applications of AuNR are the cornerstone of their biomedical applications in nanomedicine [20]. The obtained localized intense photothermal effect on exciting AuNP with a suitable laser wavelength demonstrates an immense ablation effect on cancer or bacterial cells [55,56].

**Figure 2:**
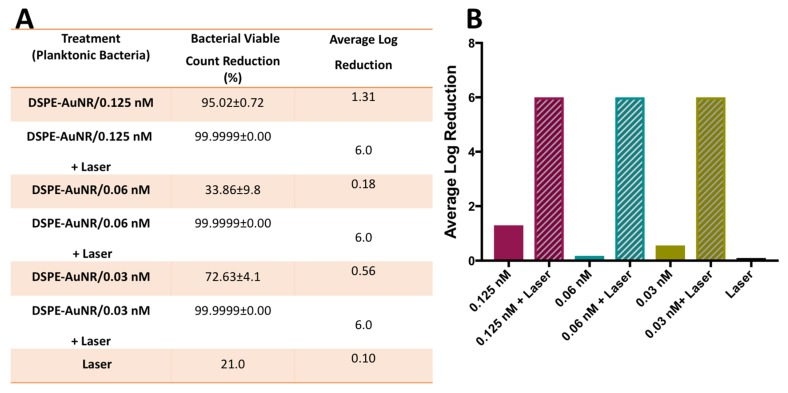
Photothermal-induced bactericidal activity of DSPE-AuNR suspension against planktonic suspension of *Pseudomonas aeruginosa*. (**A**) Calculated percentage of bacterial viable count reduction related to control upon treatment with AuNR suspension of different concentrations after NIR laser excitation (3W cm^−2^) and at dark conditions. (**B**) Calculated average log reduction of bacterial viable count upon treatment with AuNR suspension of different concentrations after NIR laser excitation and at dark conditions.

In this study, the photothermal-induced lysis or erosion activity of DSPE-AuNR against *Pseudomonas aeruginosa* was investigated at sub-MIC concentrations (0.125, 0.06 and 0.03 nM) in order to augment the antibacterial effect of AuNR at low concentrations. The results demonstrated that treatment of *Pseudomonas aeruginosa* with DSPE-AuNR suspension (0.125–0.03 nM) followed by a continuous laser beam excitation (CW, 3W cm^−2^) effected an extra ~5.0 log cycle reduction in bacterial viable count compared to the treatment using DSPE-AuNR alone (dark condition), Figure 2A,B. This synergistic effect can be related to the elevated temperature produced upon laser treatment which showed to cause areas of severe irreversible cell membrane disruption of *Pseudomonas aeruginosa* planktonic culture [25]. In earlier studies, this photothermal damage of cell membrane was attributed to multiple factors including vapor bubble formation and thermal disintegration [57].

### 2.4. Photothermal-Based Antibacterial Activity of DSPE-AuNR Suspension Against Pseudomonas Aeruginosa Biofilm

**Figure 3:**
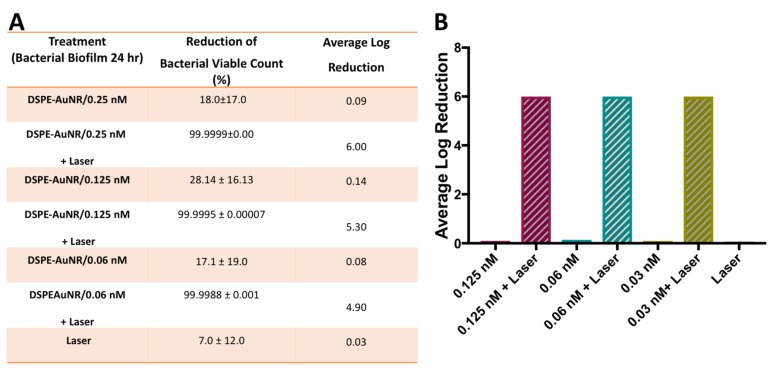
Photothermal-induced bactericidal activity of DSPE-AuNR suspension against *Pseudomonas aeruginosa* biofilm (24 h). (**A**) Calculated percentage of bacterial viable count reduction related to control upon treatment with AuNR suspension of different concentrations after NIR laser excitation (3 W cm^−2^) and at dark conditions. (**B**) Calculated average log reduction of bacterial viable count upon treatment with AuNR suspension of different concentrations after NIR laser excitation and under dark conditions.

**Figure 4:**
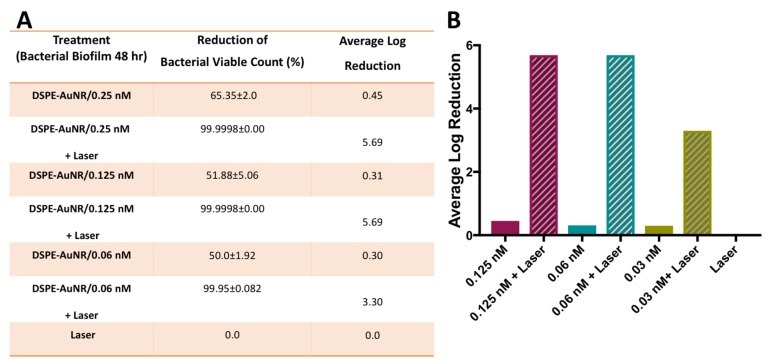
Photothermal-induced bactericidal activity of DSPE-AuNR suspension against *Pseudomonas aeruginosa* biofilm (48 h). (**A**) Calculated percentage of bacterial viable count reduction related to control on treatment with AuNR suspension of different concentrations after NIR laser excitation (3 W cm^−2^) and at dark conditions. (**B**) Calculated average log reduction of bacterial viable count on treating with AuNR suspension of different concentrations after NIR laser excitation and under dark conditions.

**Figure 5:**
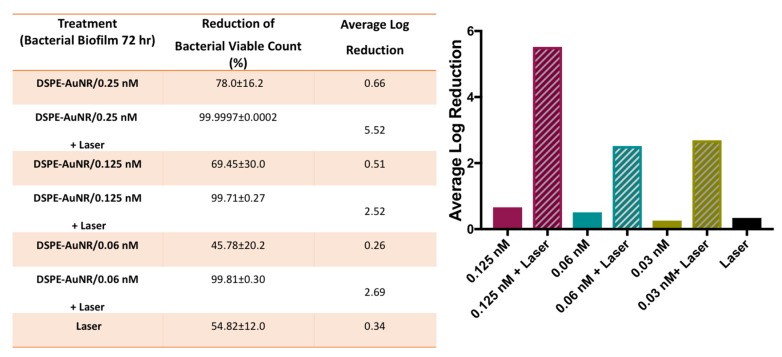
Photothermal-induced bactericidal activity of DSPE-AuNR suspension against *Pseudomonas aeruginosa* biofilm (72 h). (**A**) Calculated percentage of bacterial viable count reduction related to control upon treatment with AuNR suspension of different concentrations after NIR laser excitation (3 W cm^−2^) and at dark conditions. (**B**) Calculated average log reduction of bacterial viable count on treatment with AuNR suspension of different concentrations after NIR laser excitation and under dark conditions.

In the current study, *Pseudomonas aeruginosa* biofilms (24, 48 and 72 h) demonstrated resistance to DSPE-AuNR treatment, where it caused a non-significant decrease in viable count upon treatment under dark conditions even at the highest tested concentration; 0.25 nM (less than 78.0%, Figure 3, Figure 4 and Figure 5). Nevertheless, photothermal laser therapy unequivocally increased the killing efficiency of the DSPE-AuNR, where a significant reduction in the bacterial viable count (5.5–6.0 log reduction) was achieved after laser excitation at concentration of 0.25 nM (Figure 3, Figure 4 and Figure 5). Synchronously, lower but still significant reductions in biofilm viability were achieved at lower concentrations of excited DSPE-AuNR (Figure 3, Figure 4 and Figure 5).

In light of the reported weak antibacterial activity DSPE-AuNR on the biofilm culture, the significant antibacterial activity of our laser treated DSPE-AuNR is most probably attributed to the effect of the localized generated heat upon laser treatment. Nanoparticles interaction and penetration into biofilm involves three steps of initial transportation to biofilm vicinity followed by attachment and finally migration within the biofilm [58]. These steps are affected by the following parameters among many others; nanoparticles characteristics, biofilm matrix composition and the condition of biofilm culture including temperature. It was previously proposed that the slimy extracellular matrix of biofilm acts as an efficient sequestering agent causing significant accumulation of nanoparticles [58]. Ferry, et al., demonstrated that environmental biofilms accumulated more than 60% of added gold nanorods [59]. Additionally, nanoparticles of different physicochemical characteristics were shown to interact with and penetrate biofilms. Wan, et al., showed that a lipid shell-enveloped polymeric nanoparticle with the high integrity of lipid shells improved mucus penetration and interaction with *Pseudomonas aeruginosa* cystic fibrosis bacterial biofilm model [60]. Recently, Yu Zhao, et al., demonstrated that NIR-activated thermosensitive small positive charge spherical-shaped liposomes particles caused a combined antibiotic and photothermal therapy effect on *Pseudomonas aeruginosa* biofilm. This treatment resulted in 80% biofilm elimination at low dose and short laser excitation time [61]. Likewise, recently, Teirlinck, et al., demonstrated that laser treatment of *Pseudomonas aeruginosa* biofilms loaded with cationic 70-nm gold nanoparticles resulted in vapor nanobubbles based distortion of biofilm integrity and a subsequent improved antibiotic diffusion [62]. Accordingly, we speculate that the antibiofilm effect of our laser-activated nanoparticles is due to its interaction with the biofilm encased bacteria and the production of cytotoxic heat levels within the biofilm vicinity. Moreover, the local rise in temperature during the laser treatment session (15 min) would enhance further penetration and deposition of the DSPE-AuNR and would aid in effecting this significant ablation on the treated biofilm.

### 2.5. Photothermal-Based Antibacterial Activity of DSPE-AuNP Suspension Loaded into Poloxamer 407 Hydrogel against Pseudomonas Aeruginosa Biofilm

DSPE-AuNR were suspended in poloxamer 407 hydrogel as drug delivery for topical formulation. Thermo-responsive gels containing gold nanoparticles were used previously as antibacterial and wound healing agents, since they have the tendency to hold high water content that helps to supply dry wounds with the necessary fluids [41].

**Figure 6:**
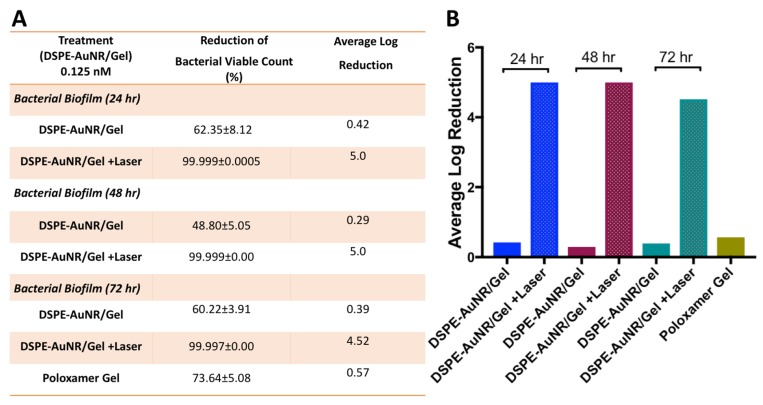
Photothermal-induced bactericidal activity of DSPE-AuNR suspension loaded into poloxamer 407 hydrogel against *Pseudomonas aeruginosa* biofilm (24, 48 & 72 h). (**A**) Calculated percentage of bacterial viable count reduction related to control upon treatment with AuNR/hydrogel after NIR laser excitation (3 W cm^−2^) and under dark conditions. (**B**) Calculated average log reduction of bacterial viable count upon treatment with AuNR/hydrogel after NIR laser excitation and under dark conditions.

Figure 6A,B demonstrate the bactericidal activity of DSPE-AuNR loaded into poloxamer 407 hydrogel against *Pseudomonas aeruginosa* biofilm (24, 48 & 72 h). Loading the nanorods into the hydrogel did not reduce their bactericidal activity. DSPE-AuNR/hydrogel happened in 4.5–5 log cycles reduction of biofilm viable count as compared to the dark condition and the hydrogel alone.

Two modes of NIR laser excitation are usually applied in the range of 700–1400 nm: pulsed or continuous (CW) modes. CW mode has been extensively used for photothermal and photodynamic therapy and commonly results in the destruction of cancer cells through hyperthermia and coagulation and may cause thermal damage to the neighbouring normal cells. Conversely, the pulsed laser is effective for selective and localized harm without causing excessive damage to the normal cells and usually results in intracellular micro bubbles; furthermore, it is known to have a cumulative effect of photothermal and photomechanical on the cells [63,64,65].

**Figure 7:**
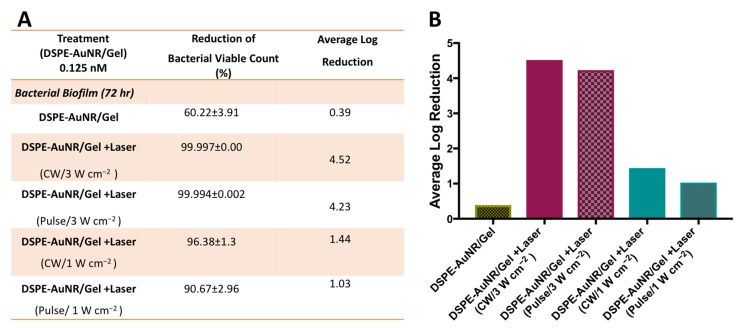
Photothermal-induced bactericidal activity of DSPE-AuNR suspension loaded into poloxamer 407 hydrogel against *Pseudomonas aeruginosa* biofilm (72 h) using CW or pulse laser at 3 W cm^−2^ and 1 W cm^−2^. (**A**) Calculated percentage of bacterial viable count reduction related to control on treatment with AuNR/hydrogel after NIR laser excitation (CW *vs*. pulse) and at dark conditions. (**B**) Calculated average log reduction of bacterial viable count on treatment with AuNR/hydrogel after NIR laser excitation (CW *vs*. pulse) and under dark conditions.

In this study, we compared the photothermal-based bactericidal activity of DSPE-AuNR/hydrogel against *Pseudomonas aeruginosa* using CW and pulse modes of laser excitation. The results indicated that utilizing different modes of laser excitation, the same percentage and log reduction of bacterial viable count were obtained: ~4 log cycle and ~1 log cycle reduction for both CW and pulse laser at 3 W cm^−2^ and 1 W cm^−2^ of the laser dose, respectively (Figure 7A,B).

**Figure 8:**
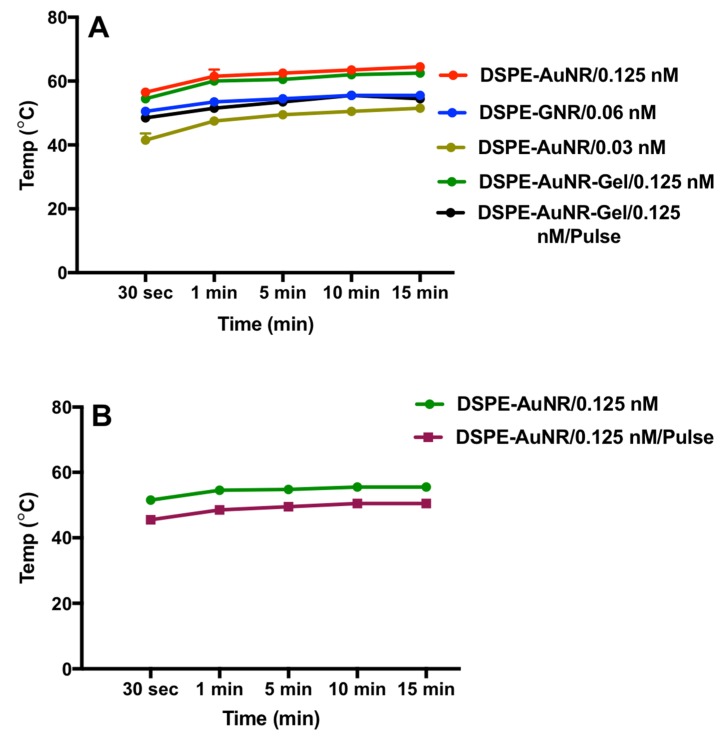
Temperature induced by NIR laser excitation of DSPE-AuNR suspensions over 15 min. (**A**) Temperature of DSPE-AuNR suspension at three different molar concentrations, DSPE-AuNR loaded into hydrogel upon excitation with CW or pulse laser (3 W cm^−2^). (**B**) Temperature of DSPE-AuNR suspension upon excitation with 1 W cm^−2^ of CW or pulse laser. The initial temperature was 19.6 ± 1.1 °C. Data are presented as mean ± SD (*n* = 3).

The localized heat obtained upon exciting DSPE-AuNR with a laser beam was recorded over 15 min of laser excitation. The colloidal stability of the nanorods was lost after 20 min of laser excitation when a significant colour change of the nanorods suspension and broadening of the longitudinal peak of UV-vis spectrum was observed (unpublished observation). The maximum temperature of the nanorods suspension upon excitation by laser was 53 °C, 60 °C and 62 °C at 0.03, 0.06 and 0.125 nM AuNR concentrations respectively. It takes one minute for the AuNR suspension to reach the maximum temperature upon laser excitation after which a slight increase in temperature was observed over time. Loading DSPE-AuNR into the hydrogel did not reduce the generated heat of the nanorods as presented in Figure 8A. Additionally, the pulse mode of the laser beam produced low maximum apparent temperature as compared to CW mode at the same laser dose (3 W cm^−2^); 55 °C *vs*. 64 °C respectively. However, both modes of laser excitation effected similar bactericidal activity. Similarly, pulse and CW modes of laser dose of 1.0 W cm^−2^ produced different apparent suspension temperature, wherein the maximum temperature was 48.5 °C and 54.0 °C respectively, after which a slight increase in temperature was observed over time (Figure 8B); however, similar bactericidal activity was obtained for both.

The pulsed laser provides extremely high and localized heat, but less average power and heat compared to the CW laser. Accordingly, favourably, the pulse laser can result in extreme temperature conditions without excessive heating of the surrounding cells and space compared to CW laser [66]. We propose that although the pulse mode of laser should lead to more intense heat generation [66], the measured heat represented an average of the heat rising and falling in times measured in nanoseconds, which consequently resulted in a lower apparent measured temperature relative to CW mode of laser beam. 

Generally, temperatures of 50 °C and above are reported to inactivate non-sporulating bacteria at a rate positively correlated with the generated heat [67,68]. For example, the thermal killing of *Pseudomonas aeruginosa* biofilm was demonstrated by thermal shock ranging from 50 °C to 80 °C for durations of 1 to 30 min, where temperature has a larger effect on biofilm eradication than exposure time [69]. Different types of materials were reported to generate heat that was sufficient to kill different types of bacteria in planktonic or biofilm cultures. Kim, et al., found that excitation of catechol-conjugated poly(vinylpyrrolidone) sulfobetaine linked to polyaniline by NIR laser caused temperature elevation (52–55 °C) that was efficient to kill *Staphylococcus aureus* and *Escherichia coli* after 3 min of NIR excitation [70]. Moreover, NIR laser excitation of graphene oxide-polyethyleneimine nanoheaters resulted in temperature elevation of more than 50 °C, that was capable to kill *Staphylococcus aureus* and *Escherichia coli* bacteria, in addition to *Staphylococcus epidermidis* bacterial biofilm [71]. Similarly, in our study, the heat generated upon DSPE-AuNP excitation is responsible for the photo-thermolysis of bacteria in planktonic or biofilm cultures; however, the possible contribution of the nanorods themselves to the observed antibacterial effect could not be excluded.

**Figure 9:**
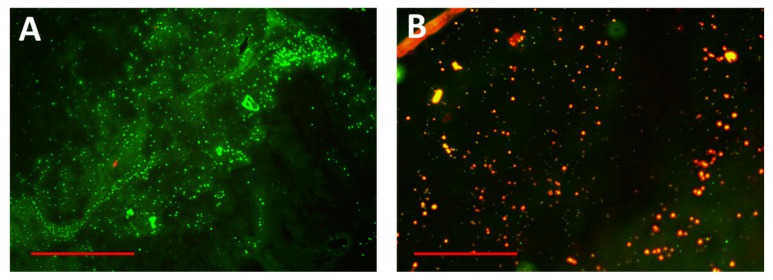
Fluorescence microscopy images of *Pseudomonas aeruginosa* biofilm on receiving photothermal therapy. (**A**) Untreated biofilm. (**B**) Biofilm treated with DSPE-AuNR and excited with a continuous laser beam. Red and green indicate dead and live bacteria respectively. Scale: 150 μm

Simultaneously, the photothermal antibiofilm effect was observed by fluorescent microscopy [71]. Fluorescence microscopy imaging of the bacterial biofilm after staining with live-dead stain revealed that exciting the bacterial biofilm with a laser beam after treatment with DSPE-AuNR resulted in significant death of biofilm cells (Figure 9A) compared to the control (Figure 9B). These results confirm the role of photothermal therapy in eradicating the bacterial biofilm using surface modified gold nanorods.

**Figure 10:**
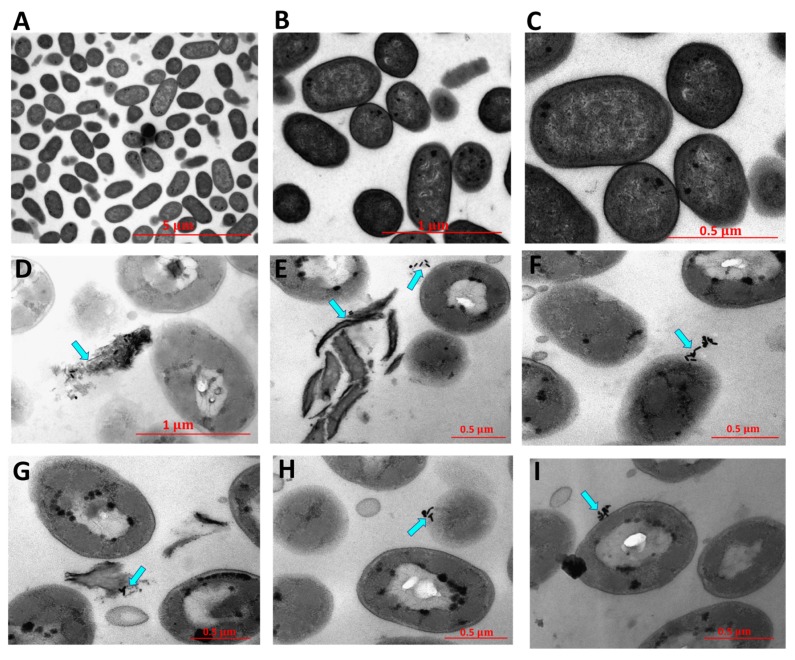
TEM images of untreated *Pseudomonas aeruginosa* (**A**–**C**) and after treatment with DSPE-AuNR and excitation with CW laser beam (**D**–**I**). Photothermal therapy resulted in significant changes in the morphology of the bacteria and lysis of bacterial cells.

The photothermal-induced bactericidal activity of DSPE-AuNP against *Pseudomonas aeruginosa* was also investigated by TEM imaging. Untreated bacteria showed well-defined intact rod-shape cells (Figure 10A–C). Upon treatment with DSPE-AuNR and excitation with laser, bacteria demonstrated significant changes in their shape, drastic disintegration of the bacterial membrane and complete lysis of the bacteria (Figure 10D–I). These findings confirm our hypothesis of the ability of DSPE-AuNR to penetrate the bacterial cell membrane and maximize the photothermal-induced destruction and lysis of the cells upon laser excitation.

## 3. Materials and Methods

### 3.1. Chemical Synthesis of AuNR Suspension

Synthesis of AuNP was carried out using CTAB (Sigma-Aldrich Chemicals, St. Louis, MO, USA) following a method described previously [42]. Briefly, for seed synthesis, a solution of HAuCl4 (0.25 mM) was prepared in 0.1 M CTAB. A volume of 0.6 mL of NaBH_4_ was added to gold-CTAB solution (10.0 mL) under stirring. Subsequently, the following materials were added to 95.0 mL of CTAB aqueous solution (0.1 M) for AuNR synthesis: 5.0 mL of HAuCl_4_ solution (10 mM), 1.2 mL of silver nitrate solution (10.0 mM), 0.55 mL of ascorbic acid solution (0.1 M) and 0.12 mL of the seed solution. AuNR suspension was left overnight at 25 °C overnight. The obtained AuNP suspension was centrifuged twice (Centrifuge, Labofuge I, Heraeus Christ, Hanau, Germany) for 10 min at 16,500× *g*, suspended in milli-Q water and stored at 4 °C.

### 3.2. Surface Modification of AuNR with DSPE-PEG-SH (DSPE-PEG-SH); DSPE-AuNR

A volume of 1.0 mL of DSPE-PEG-SH solution (25.0 mg/mL, MW ~2000 g/mole, Nanosoft Polymers, Winston-Salem, NC, USA) was mixed with AuNP suspension (10.0 mL) and kept under stirring condition for 24 h. The resulted surface-modified AuNP suspension was centrifuged twice for 10 min at 11510× *g* in order to remove excess CTAB and was stored at 4 °C.

### 3.3. Characterization of AuNR and DSPE-AuNR Suspensions

The prepared AuNP and DSPE-AuNR suspensions were characterized by UV-vis absorption spectra (UV-Vis Spectrophotometer, Shimadzu UV-1800, Kyoto, Japan), zeta potential (Nicomp Nano Z3000 particle size/zeta potential analyzer (Particle Sizing Systems, Santa Barbara, CA, USA) and TEM imaging (FEI Morgani 268, operating voltage of 60 kV, Eindhoven, The Netherlands). The surface modification of AuNR by DSPE-PEG-SH ligand was confirmed by proton nuclear magnetic resonance (^1^H NMR) in our recent work [22].

### 3.4. Loading of DSPE-AuNR into Poloxamer 407 Hydrogel

DSPE-AuNR suspensions of different concentrations were loaded into poloxamer 407 polymer (18%, BSAF, Ludwigshafen, Germany). The obtained hydrogels were kept at 4 °C for 24 h, and then were sterilized by UV light for 45 min before use.

The obtained DSPE-AuNR/hydrogels were characterized by their colloidal colour, UV-Vis absorption spectra and zeta potential. The sol-gel transition temperature of the hydrogel of DSPE-AuNR was measured following the method described previously [72].

### 3.5. Determination of the Bacteriostatic and Bactericidal Activities of DSPE-AuNR Suspension Against Pseudomonas Aeruginosa

#### 3.5.1. Bacterial Strains

Overnight working cultures of *Pseudomonas aeruginosa,* ATCC 9027 (Oxoid, Basingstoke-Hampshire, UK) grown in nutrient broth medium (Oxoid, Basingstoke, UK), adjusted to 0.5 McFarland standard were used throughout the study. Long term preservation of the cultures was done at −80 °C with glycerol, while short term preservation was done on nutrient agar plates at 4 °C.

The bacteriostatic activity of DSPE-AuNP suspension was determined using the standard two-fold broth micro dilution method which determines the minimal inhibitory concentration (MIC) of DSPE-AuNP against *Pseudomonas aeruginosa* planktonic cultures according to the Clinical and Laboratory Standards Institute (CLSI, 2016).

Briefly, wells of 96 microtiter plates (JET BIOFIL, Guangzhou, China) were filled with 150 μL of Muller Hinton broth (Oxoid, UK). The first well was filled with a double strength media. To the first well, 150 μL of DSPE-AuNP stock suspension (4 nM) was added, from which 10 two-fold serial dilutions were made across the plate’s well prefilled with 150 μL of single strength media. Each well was subsequently inoculated with a MacFarland-adjusted overnight culture to obtain an inoculum size of *ca*. 1 × 10^6^ CFU/mL in each well. For each plate set up, positive and negative controls were used. Plates were incubated overnight aerobically at 37 °C. After incubation, the MIC value was determined as the average of DSPE-AuNR concentrations that caused inhibition and no inhibition within the sequentially ordered wells. Subsequently, the number of bacterial cells (CFU/mL) within the well of lowest concentration exhibiting no turbidity was determined using the standard spread plate count method as performed previously [21]. To do that, a volume of 100 μL of the designated well was serially ten-fold diluted in normal saline. A volume of 100 μL of each dilution was spread onto nutrient agar plates (Oxoid, Basingstoke, UK). The plates were incubated overnight aerobically at 37 °C.

The number of bacterial colonies and the dilution factor were used to calculate the viable bacterial cells (CFU/mL). These values were compared to the initial inoculum size to calculate the percent reduction in the viable count and the log reduction in viability as a measure of the bactericidal activity of DSPE-AuNR suspension.

In order to exclude the possible contribution of residual CTAB or the coating material to the observed antibacterial of AuNR, the MIC test was performed for the supernatant of DSPE-AuNR after centrifugation twice for 10 min at 11510× *g* and for the aqueous dispersion of DSPE-PEG-SH ligand.

#### 3.5.2. Photothermic Ablation Activity of DSPE-AuNR Suspension against *Pseudomonas aeruginosa* Planktonic Culture

The photothermal-bactericidal activity of DSPE-AuNR upon laser treatment to *Pseudomonas aeruginosa* planktonic culture was determined by exciting a pre-incubated (10 min) inoculated DSPE-AuNR suspension with CW 808 nm, ~1–3 W cm^−2^, or pulse mode, pulse width of ~1.0 μs at area of ~0.5 cm^2^ (Laser diode, max power output 9 W, 808 nm, OSTECH, Berlin, Germany) for 15 min. To do that, 150 μL of different concentrations of DSPE-AuNP suspension (0.25, 0.125, 0.06. 0.03 nM) and 10 μL of standardized culture were added (*ca*. 1 × 10^6^ CFU/mL) to 150 μL of single strength media. This set up was incubated at 37 °C for 10 min. Subsequently, it was excited using either continuous laser or pulse laser mode for 15 min. The photothermal-based bactericidal activity of DSPE-AuNR suspension was determined using the standard plate count method described previously.

The temperature of DSPE-AuNR suspensions exposed to different laser intensities was measured by inserting a digital thermometer probe (Extech, FLIR Commercial Systems Inc., Wilsonville, OR, USA) into the suspensions. The temperature of ultrapure water exposed to laser excitation was also measured and subtracted from that of the AuNR solutions. The initial temperature of the AuNR suspensions was recorded as well.

Non-laser treated replicate of the above mentioned set up was used as the dark control (25 min of incubation at 37 °C). Light control was prepared by exposing the culture to a laser beam for 15 min using the same photothermic parameters as the test experiments.

#### 3.5.3. Biofilm Culture of *Pseudomonas aeruginosa*

Biofilms were cultured on sterile borosilicate glass beads with a diameter range of 3–3.5 mm. Each bead was placed in a well of a 96-well microtiter plate (JET BIOFIL, Guangzhou, China), to which 200 μL of nutrient broth was added, followed by 20 μL of a standardized *Pseudomonas aeruginosa* culture to obtain an inoculum size of *ca*. 1 × 10^6^ CFU/mL. The plates were incubated aerobically at 37 °C for 72 h in a shaker incubator. After every 24 h, the spent media was pipetted out carefully and replaced by 200 μL of fresh media. The density of biofilm formed at 24, 48 and 72 h was determined using the standard plate count method and expressed as CFU/bead.

After biofilm culturing, beads were removed and washed three times using normal saline to remove loosely attached bacterial cells. Each bead was placed in a centrifuge tube filled with normal saline (100 μL), vortexed to remove attached bacteria, serially diluted over five ten-fold serial dilutions and then plated out (100 μL) on nutrient agar media. The number of colonies and dilution factors were used to quantitatively determine biofilm density as CFU/bead.

#### 3.5.4. Photothermal-Induced Antibiofilm Activity of DSPE-AuNR Suspension

The bactericidal activity of DSPE-AuNR suspension (0.25, 0.125, 0.06 and 0.03 nM) on 24, 48 and 72 h biofilm cultures was determined by challenging the washed biofilm beads with DSPE-AuNR suspension for 10 min incubation at 37 °C, followed by laser excitation for 15 min using continuous or pulse mode.

After treatment, beads were recovered, and biofilm viable density was determined following the method previously mentioned. The antibiofilm activity was compared to the positive control of untreated biofilm and the dark control (25 min of incubation at 37 °C). The percentage reduction in viable count and log reduction in viability were calculated.

#### 3.5.5. Photothermic Ablation Activity of DSPE-AuNR/Gel against *Pseudomonas aeruginosa* Biofilm Culture

The photothermic ablation effect of 0.125 DSPE-AuNR/Gel on the viability of 72-h biofilms under multiple laser excitation conditions was determined using the viable count method described previously.

The washed pre-incubated (10 min) biofilm was excited by continuous or pulsed laser for 15 min. Post treatment, the bactericidal activity was determined, and the ablation effect was calculated as percent killing or log reduction relative to the untreated biofilm and compared to the dark control (25 min of incubation at 37 °C).

#### 3.5.6. Characterization of Biofilm Viability by Fluorescent Microscopy

Filmtracer LIVE/DEAD biofilm viability kit (Invitrogen, Thermo Fischer Scientific, Eugene, OR, USA) was used to stain the biofilm. A 72-h biofilm was prepared on micro cover glasses (Deckgläser, Schwerte, Germany) in 12 well cell culture plate (SPL, Korea). The wells were filled with 2 mL nutrient broth (Oxoid, Basingstoke, UK). Each well was inoculated with *ca*. 1 × 10^6^ CFU/mL of overnight culture of *Pseudomonas aeruginosa*. The culture set up was incubated in shaker incubator for 72 h. After every 24 h, the media was changed and replaced with fresh media. For staining, the 72-h biofilm was washed three times with filter sterilized water. To each glass cover, 200 µL of the dead/live stain was added. The setup was incubated away from light at room temperature for 25 min. Subsequently, the micro cover glasses were gently rinsed with filter-sterilized water. All the excess stain and rinse water were removed from the base of the well. The coverslips were placed on glass slides and were imaged using EVOS M5000 cell imaging system (Invitrogen, Thermo Fischer Scientific, Eugene, OR, USA) at 20× using EVOS GFP light Cube (AMFEP4651; excitation 470/22 nm; emission 510/42 nm) and EVOS RFP Light Cube (AMEP4652; excitation 531/40; emission 593/40 nm).

#### 3.5.7. Characterization of Photothermal Ablation Activity of DSPE-AuNR Suspension against *Pseudomonas aeruginosa* Biofilm by Transmission Electron Microscope (TEM)

An overnight culture of *Pseudomonas aeruginosa* (250 μL, *ca*. 1.0 × 10^8^ CFU/mL) was mixed with DSPE-AuNR (50 μL, 0.25 nM) and was incubated for 10 min at 37 °C. The mixture was exposed to a laser beam (808 nm, CW, 3W cm^−2^) for 15 min; then, the mixture was pelleted at 6000 rpm for 10 min and the obtained bacterial pellets were fixed in 3% glutaraldehyde (Sigma-Aldrich Chemicals, St. Louis, MO, USA) and processed as described previously [21]. Sections approximately 70 nm thick were obtained and mounted onto Formvar copper grids and imaged by TEM. Untreated bacteria were processed as previously described and was used as a control.

## 4. Conclusions

The eradication of *Pseudomonas aeruginosa* biofilm is becoming a challenge in the treatment of several skin infections. The photothermal ablation activity of non-spherical shapes of gold nanoparticles particularly nanorods is the cornerstone of nanomedicine. In this study, we shed light on the ability of gold nanorods decorated with phospholipid to destroy *Pseudomonas aeruginosa* biofilm in vitro. The surface modification of gold nanorods with a phospholipid bilayer resulted in the enhancement of their uptake in the bacterial cell membrane and maximization of photothermal ablation activity when excited with a continuous or pulse laser beam. The TEM imaging of the photothermally-treated cells indicated a massive disintegration of bacterial cell membrane and subsequent cell lysis compared to the untreated cells. Loading the gold nanorods into poloxamer 407 hydrogel did not reduce their bactericidal activity upon excitation with a laser beam. A gold-based nano system could be considered an effective alternative to antibiotics to eradicate the biofilm of *Pseudomonas aeruginosa.*
